# The infective cycle of Cabbage leaf curl virus (CaLCuV) is affected by CRUMPLED LEAF (CRL) gene in Arabidopsis thaliana

**DOI:** 10.1186/1743-422X-6-169

**Published:** 2009-10-20

**Authors:** Diana L Trejo-Saavedra, Jean P Vielle-Calzada, Rafael F Rivera-Bustamante

**Affiliations:** 1 Departamento de Ingeniería Genética, Centro de Investigación y de Estudios Avanzados del IPN (Cinvestav), Unidad Irapuato, Km. 9.6 Libramiento Norte, P.O. Box 629, C.P. 36500, Irapuato, Guanajuato, México

## Abstract

**Background:**

Geminiviruses are single-stranded DNA viruses that cause serious crop losses worldwide. Successful infection by these pathogens depends extensively on virus-host intermolecular interactions that allow them to express their gene products, to replicate their genomes and to move to adjacent cells and throughout the plant.

**Results:**

To identify host genes that show an altered regulation in response to *Cabbage leaf curl virus *(CaLCuV) infection, a screening of transposant *Arabidopsis thaliana *lines was carried out. Several genes were identified to be virus responsive and one, *Crumpled leaf *(*CRL) *gene, was selected for further characterization. *CRL *was previously reported by Asano et al., (2004) to affect the morphogenesis of all plant organs and the division of plastids. We report here that *CRL *expression, during CaLCuV infection, shows a short but strong induction at an early stage (3-5 days post inoculation, dpi). To study the role of *CRL *in CaLCuV infection, *CRL *over-expressing and silenced transgenic plants were generated. We compared the replication, movement and infectivity of CaLCuV in transgenic and wild type plants.

**Conclusion:**

Our results showed that CRL over-expressing plants showed an increased susceptibility to CaLCuV infection (as compared to wt plants) whereas *CRL*-silenced plants, on the contrary, presented a reduced susceptibility to viral infection. The possible role of *CRL *in the CaLCuV infection cycle is discussed.

## Background

Geminiviruses are a large and diverse family of plant viruses that are packed as single-stranded, circular DNA genomes and characterized by virions (or capsides) with twin icosahedral morphology and a replication process based on rolling circle and recombination-dependent mechanisms [1-3]. The family *Geminiviridae *is taxonomically divided in four genera according to their range, insect vector, phylogenetic relatedness and genome organization (mono- or bipartite) [4]. The genus *Begomovirus *is the largest one and comprises all whitefly-transmitted geminiviruses that infect dicotyledonous plants [5]. DNA A of a typical bipartite begomovirus encodes four/five proteins: Rep and REn involved in replication; AC4, encoding a suppressor of silencing; TrAP involved in transcriptional activation and silencing suppression and CP, the coat protein. DNA B encodes the movement protein (MP) and the nuclear shuttle protein (NSP), both required for systemic infection [4,5]. *Cabbage leaf curl virus *(CaLCuV) is a bipartite begomovirus that infects a broad range of members of the *Brassicaceae *including cabbage, cauliflower and *Arabidopsis thaliana *[6].

Successful infection by a geminivirus, as any other virus, depends on its ability to express its gene products, to replicate its genome and to move to adjacent cells. Accordingly, the geminivirus infection cycle has been shown to depend extensively on virus-host intermolecular interactions, which are required either for basic compatibility or for modulation of virus infection by subverting defense responses [7]. In two cases, virus replication has been reported in differentiated cells, which do not contain detectable levels of replicative polymerases, and, therefore, should not be competent for DNA replication [8,9]. Consequently, it has been suggested that an early step in a geminivirus infection process is a reprogramming of plant cell-cycle controls to induce the synthesis of the host DNA replication machinery [10,11]. Viral movement requires the action of virus-encoded movement proteins to coordinate the replication of the viral genome with its cell-to-cell transport [12,13]. The nuclear localization of geminivirus during replication implies that two movement steps must be achieved for systemic infection, one to move the viral genome from the nucleus to the cytoplasm and then, another one to allow a cell-to-cell movement across the cell walls [12,14].

According to current models, viral infection affects the expression of many plant genes both temporally and spatially [15]. Some genes may show altered patterns of gene expression in response to virus infection due to the activation of the defense mechanisms against the invading pathogen [16,17]. In addition, changes in host gene expression may also occur when cellular functions are redirected to support the synthesis of viral nucleic acids and proteins, resulting in changes in plant metabolism and often, the development of symptoms [18,19]. Research on plant virus-host interactions is currently providing considerable insights into the mechanism by which viruses interact with host proteins. Several proteins are known to be interacting with viral proteins in the infected cell. For example, it has been shown that Rep from *Tomato golden mosaic virus *(TGMV) interacts with the proliferating cell nuclear antigen (PCNA) and the cell cycle regulator retinoblastoma (pRB) in order to reprogram the host cell cycle to create a replication-competent environment [20]. In the case of *Wheat dwarf virus *(WDV), it has been suggested that Rep interacts with the replication factor C (RFC). This interaction may represent an early step in the assembly of an elongation complex during geminivirus DNA-primed DNA replication [21].

Most of the proteins known to interact with viral proteins have been identified using two hybrid systems or pull-down assays [21-24]. These interactions do not identify those genes that are up or down-regulated as a consequence of a viral infection, but whose products do not necessarily directly interact with viral proteins. That is the reason why a better understanding of the transcriptional changes occurring during the initial events of a virus infection could provide relevant insights into how plants recognize and respond to viruses, and how these pathogens cause disease.

Microarray analyses have been shown to be a powerful methodology to identify host genes whose expression is altered during an infection by a geminivirus [25]. Nevertheless, there are cases in which affected genes would not be detected because a dilution effect due to a highly localized expression, a low number of infected cells, or even due to an inappropriate timing for RNA sample collection.

In an attempt to identify host genes whose expression is modified in the early events of a geminiviral infection, we screened an *Arabidopsis thaliana *collection transformed with transposon-based, enhancer- or gene-trap vectors (MET or MGT). The enhancer-/gene-trap elements carry a reporter gene construct that can respond to *cis*-acting transcriptional signal at the insertion site [26-28]. These elements permit the identification of genes by their pattern of expression and their subsequent cloning using the inserted element as a tag [26]. This system can be adapted easily to a large scale for identification of pathogen responsive genes.

Using this methodology we have identified a sequence corresponding to the *Crumpled Leaf *gene (*CRL*). A *crl *mutant exhibits a symptom-like phenotype similar to the one observed in geminivirus-infected *A. thaliana*: dwarfing, chlorotic mottle, yellow mosaic and crumpled leaves [29].

We analyzed *CRL *expression in wild-type infected plants and used RNA-interference methodology (RNAi) and ectopic expression in Arabidopsis as efficient forward genetic approaches to analyze the function of the *CRL *gene. Results suggest that *CRL *is involved in the infective process since altering CRL levels altered susceptibility to CaLCuV infection.

## Results

### Screening and identification of virus-infection inducible genes

A total of 506 transposant lines were analyzed: 273 MGT and 233 MET lines. For each line, a total of 40 seeds were germinated at 22°C in a controlled environment chamber. Plants at 6-8 leaves stage were inoculated with CaLCuV DNA in a single leaf using a hand-held biolistic device. As wound control for the biolistic inoculation, plants from each line were mock-inoculated with empty-plasmid DNA or with gold particles with no DNA. The effect of the inoculation was studied by excising bombarded leaves at 1, 3, 5, and 7 days post-inoculation (dpi) to be assayed for GUS activity by histochemical staining (6 plants per assay).

After the assays, 11 lines that showed an up-regulation of GUS expression after virus inoculation (but not after a mock inoculation control) were selected for further analysis. Ten of these lines were from the gene-trap collection whereas only 1 line was from the enhancer-trap collection. The remaining 495 lines included those that did not show GUS expression at all (306 lines), lines that did not change their GUS expression patterns after infection (168 lines), or lines that showed an altered GUS expression due to a wound-response (i.e., responsive to both, virus- and mock-inoculation, 22 lines).

To identify the insertion site of the transposon and, therefore, the tagged genes, thermal asymmetric interlaced (TAIL)-PCR was performed as described [30]. After the identification of tagged genes, the *CRUMPLED LEAF *(*CRL*)-tagging line (MGT 208) was selected for further analysis since a *crl *mutant exhibits an interesting phenotype that resembles, in a mild form, the symptoms observed in virus-infected plants. In addition, no correlation with a biotic stress has been reported for this gene [29,31]. An extended analysis of the gene/enhancer trap strategy to identify pathogen-related genes, the protocol for the screening and the type of identified tagged genes responsive to geminivirus infection is being presented elsewhere (Trejo-Saavedra *et al*., in preparation).

### *CRL *expression is modified by viral DNA

**Figure 1:**
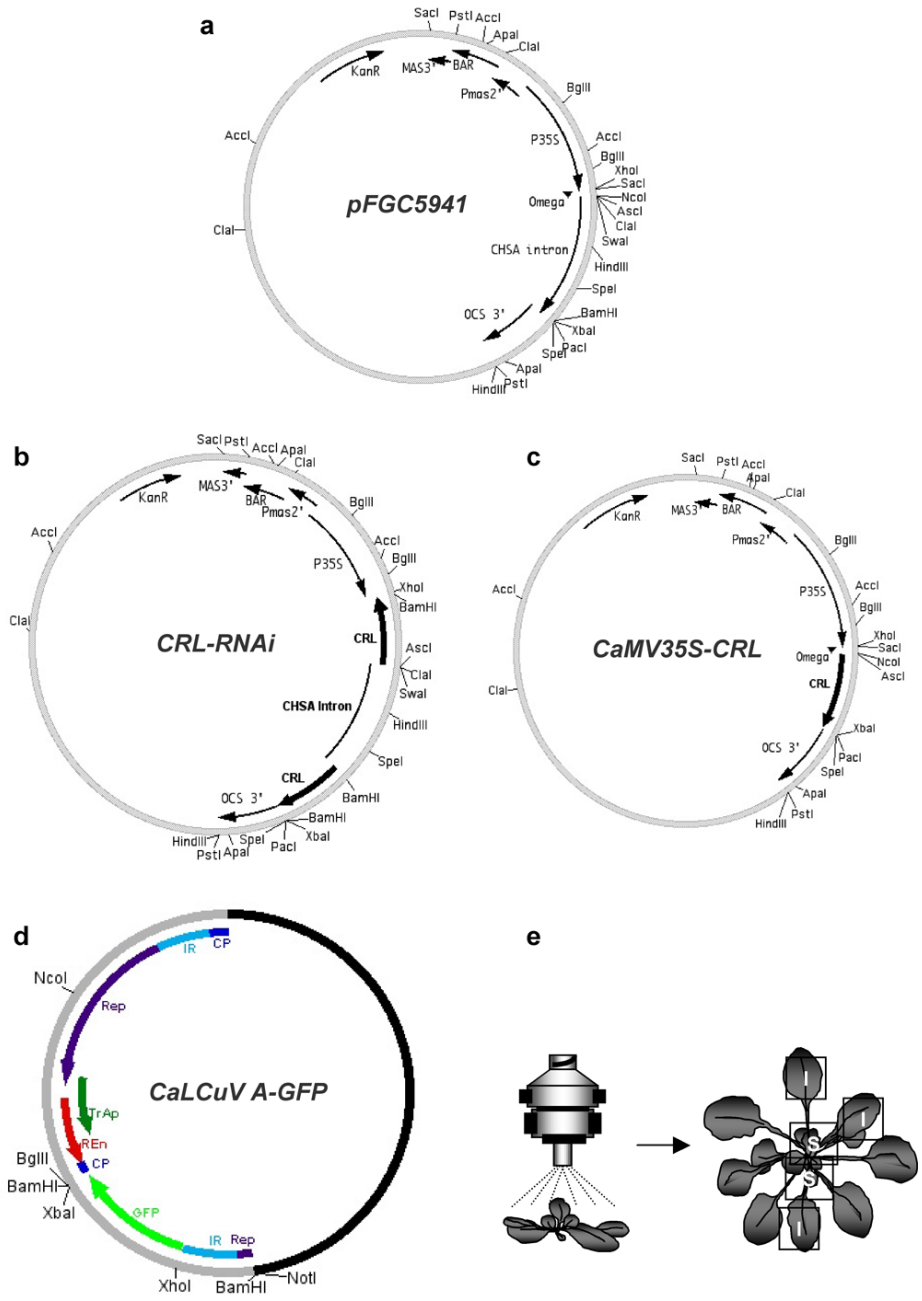
**Map of plasmid pFGC5941, *CRL *vectors, CaLCuV A-GFP construct and viral-inoculation method**. **a**, Map of plasmid pFGC5941 indicating 35S promoter, CHSA intron sequence and restriction sites. **b**, Map of CRL-RNAi construct indicating *CRL *open read frames cloned in both senses. **c**, Map of CaMV35S-CRL construct indicating *CRL *open read frame cloned downstream of 35S promoter. **d**, Genomic map of CaLCuV A-GFP construct indicating GFP open reading frame and restriction sites. **e**, Virus inoculation method indicating the inoculated (I) and systemic (S) tissues.

The expression of *CRL *in CaLCuV-infected plants was analyzed by real-time RT-PCR. *Arabidopsis thaliana *plants at the 6-8 leaves stage were inoculated with CaLCuV DNA by a biolistic procedure (Figure 1e). Inoculated and systemic leaves (leaves that appeared after the inoculation) were collected at 1, 3, 5 and 7 days post inoculation (dpi). Total RNA was extracted from leaves and compared with similar leaves from two types of control plants: mock-inoculated and untreated (not bombarded) plants. To eliminate possible contamination by genomic DNA, PCR primers were designed to be located in different exons; therefore, the PCR product size indicates the type of template (DNA or RNA) used by the polymerase. The results were normalized using a parallel RT-PCR assay for 16S rRNA.

**Figure 2:**
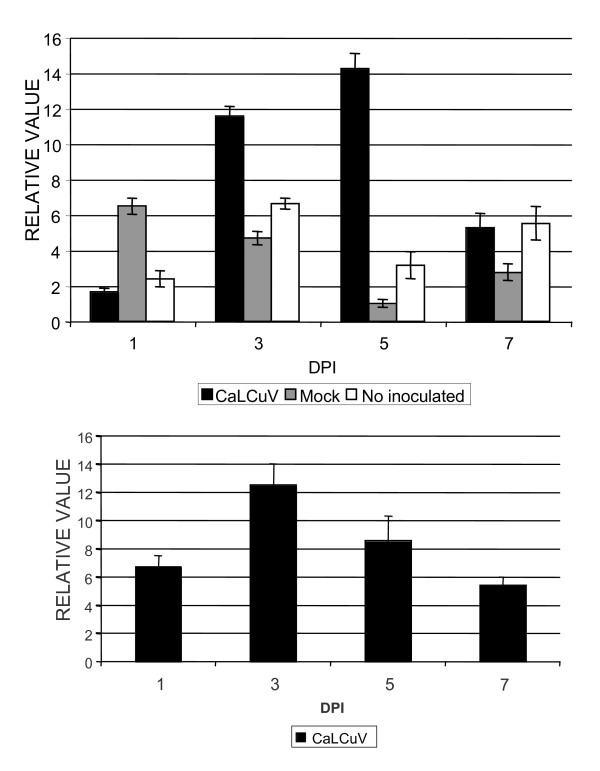
**Relative level of *CRL *and CaLCuV transcripts**. **a**, Relative level of *CLR *transcript in virus-, mock- and not-inoculated wild-type Arabidopsis plants at different dpi, measured by real-time RT-PCR. **b**, Relative level of CaLCuV in virus-inoculated wild-type Arabidopsis plants at different dpi. Each bar corresponds to the mean value from leaves of five plants. Standard deviation is also included.

As seen in figure 2a, the concentration of *CRL *RNA increases after infection with a peak around 5 dpi and a decrease to basal levels at 7-9 dpi. In an attempt to corroborate this expression, 11 independent, transgenic lines containing a 883 bp version of CRL promoter (883 nt) fused to GUS marker gene (pCRL::UidA lines) were obtained. Unfortunately, with this promoter version, the GUS expression in all plants (inoculated and non inoculated controls) was relatively high, thus, it was rather difficult to appreciate differences between treatments using this histochemical procedure (data not shown). To corroborate the expression of *CRL *and the presence of the virus, a parallel viral RNA assay was also performed. Figure 2b, shows the RT-PCR quantification of viral RNA using primers located inside the rep ORF. Interestingly, both RNA types, viral and CRL, showed a similar pattern. However, the peak of viral RNA concentration seems to precede the peak of CRL RNA, suggesting a cause-effect relationship.

### Generation of CRL-expressing (*35S-CRL*) and silenced (*CRL-RNAi*) lines

**Figure 3:**
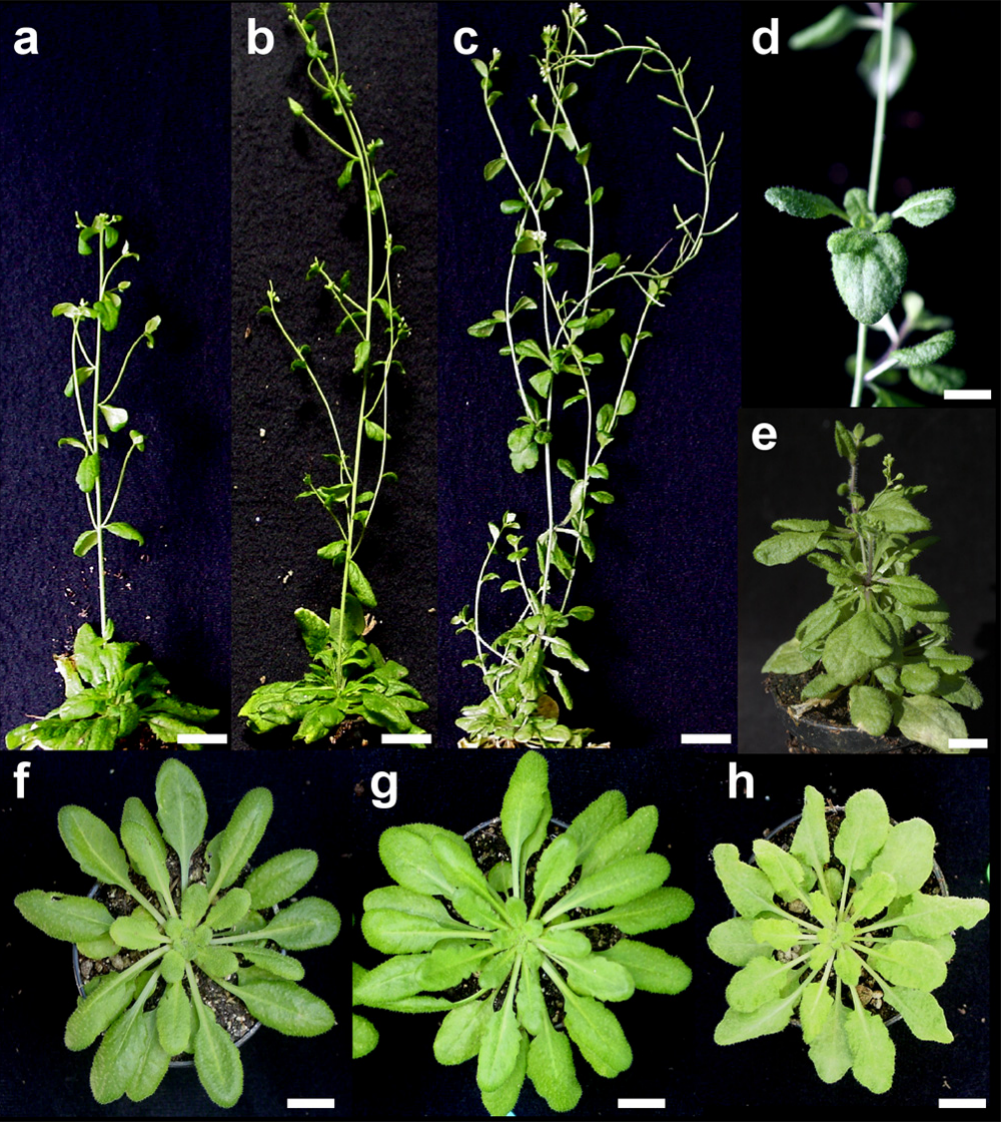
**Phenotype of transgenic lines**. **a**, Control wild-type plant; **b**, Transgenic *CaMV35S-CRL *T2-1 adult plant; **c**, Transgenic *CRL-RNAi *T2-5 adult plant showing typical strong phenotype; **d **and **e**, Inflorescence of a *CRL-RNAi *plant showing development of small rosettes instead of flowers; **f**, Rosette of a wild-type plant; **g**, Rosette of a *CaMV35S-CRL *T2-1 transgenic plant; **h**, Rosette of a *CRL-RNAi *T2-5 transgenic plant showing a reduced diameter and pale crumpled leaves. Bars = 3 cm (a-c), 1 cm (d-h).

To study the involvement of *CRL *in CaLCuV infection, we carried out experiments based on loss- and gain-of-function strategies. For the loss-of-function assays, we silenced the *CRL *gene in Arabidopsis plants making a *CRL*-RNAi construct. The complete *CRL *ORF was cloned in both orientations into a pFGC5941 RNAi vector that contains the strong 35S promoter of the *Cauliflower mosaic virus *(CaMV35S) (Figure 1b). The vector was then used to transform wild-type Col-0 plants. A total of 45 primary transformant plants were generated, showing different classes of phenotypes. Previous studies targeting endogenous genes with similar RNAi strategies have been shown to produce a series of mutant phenotypes that vary from weak phenotypes to phenotypes resembling known null mutants of the targeted gene [32-35]. The 45 lines were grouped into three classes: 20 lines (44.4%) showed no altered phenotype. Eight lines (17.7%) showed a weak phenotype that consisted of a slightly crumpled leaf phenotype. The final group of 17 plants (37.7%) showed crumpled leaves, dwarf plant and pale green phenotype (Figure 3), consistent with the previously reported null mutant [29]. It is important to mention that the phenotypes observed in the silenced lines were clearly distinguishable from the symptoms induced by CaLCuV as discussed below. Interestingly, under certain conditions (8 h light/16 h dark photoperiod, 24 C), the last group of plants also showed an additional phenotype. Small rosette-like structures were observed instead of flowering stems. New stems developed from those rosettes (Figure 3d, e) confirming the equivalence of the structure. This phenotype, not observed under normal greenhouse conditions, was maintained in the T2 generation. This additional phenotype was not reported with the null mutant probably due to the requirement of a short photoperiod condition [29]. More recently, it has been shown that *crl *mutant Arabidopsis plants contain cells lacking detectable plastids [31]. The alteration in the generation of cells lacking plastids might be responsible for the phenotype observed.

For the gain-of-function approach, we over-expressed *CRL *gene using a modified version of the pFGC5941 vector that contained the CaMV35S promoter directing the sense expression of *CRL *ORF sequence (Figure 1c). A total of 61 over-expressing primary transformed plants were generated. All lines showed an intense green colour when compared with the wild type (Figure 3b, g), however, no additional morphological differences were observed between wild type and *CaMV35S-CRL *lines.

**Figure 4:**
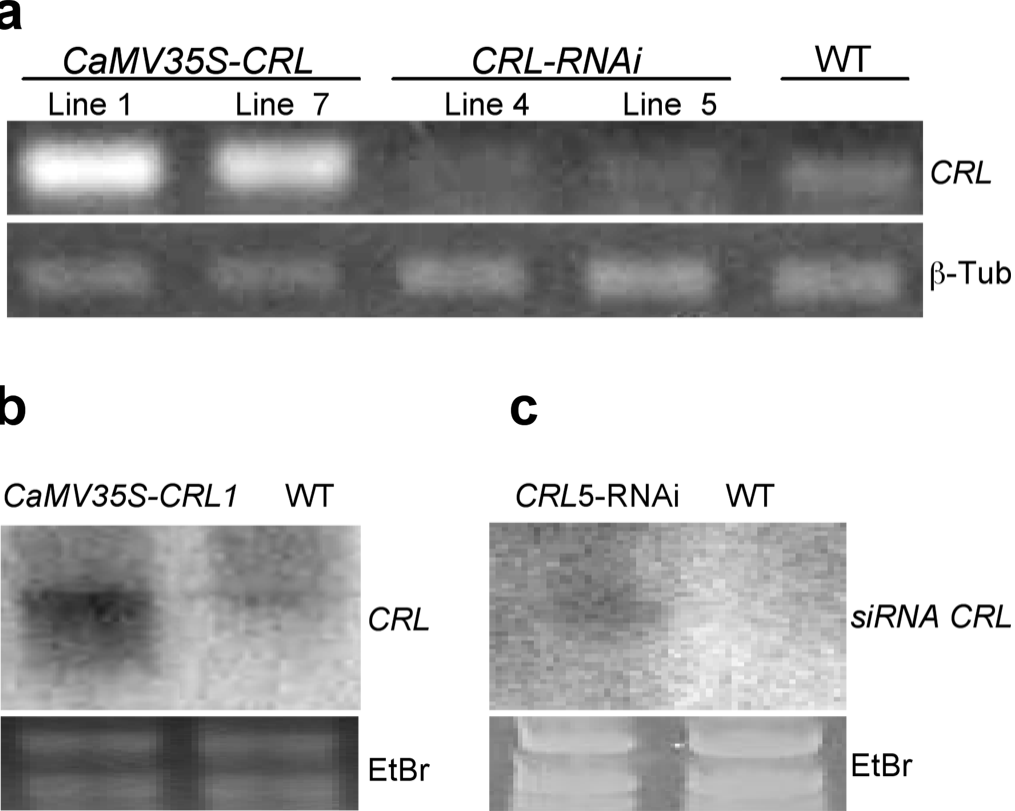
**Accumulation of *CRL *transcripts in *CaMV35S-CRL *and *CRL-RNAi *T2 lines**. **a**, RT-PCR showing increased levels of *CRL *transcripts in *CaMV35S-CRL *T2-1 and T2-7 plants, contrasting with decreased levels in *CRL-RNAi *T2-4 and T2-5 as compared with levels from a wild-type plant. **b**, Expression analysis of *CaMV35S-CRL *T2-1 and wild-type control by northern blot. **c**, Small interfering RNA northern blot analysis of *CRL-RNAi *T2-5 line and wild-type plants. In all cases RNA was isolated from rosettes at 30 days post-germination. For **b **and **c**, complete *CRL *cDNA was used as probe. Ethidium bromide staining of rRNAs is shown as loading control.

To corroborate the relationship between the observed phenotypes and the level of *CRL *transcript, total RNA was extracted from rosette leaves from two independent lines from each strategy: *CaMV35S-CRL *and *CRL-RNAi*. In the case of the silenced lines, we selected plants (T2) showing a strong phenotype. Figure 4 shows the results of an RT-PCR analysis of CRL transcripts in both cases. Compared to wild type (wt) plants, the analyzed *CaMV35S-CRL *lines (T2-1 and T2-7) indeed showed a substantial increase in *CRL *transcript levels. On the other hand, the *CRL-RNAi *lines (T2-4 and T2-5), which exhibited a strong phenotype, showed reduced transcript levels (Figure 4a). A northern blot analysis was also performed to corroborate the altered expression (Figure 4b). The presence of small interfering RNAs (siRNA, 21-23 nt long) has been suggested as a hallmark of a silencing process affecting a given gene. Therefore, the presence of siRNAs related to *CRL *was analyzed in RNA extracts from silenced and wt lines. The results are also shown in figure 4. β-tubulin was used as a constitutive control to show that equal amounts of RNA were used. *CRL*-related siRNAs were detected in RNA extracts from line *CRL-RNAi *T2-5 line but not in those from wt plant (Figure 4c), suggesting the degradation of the *CRL *transcript by RNA silencing mechanism [36].

For further analysis, we selected T2 plants from lines *CaMV35S-CRL *T2-1 line (strong expression of *CRL *gene, no phenotype) and *CRL-RNAi *T2-5 (no *CRL *mRNA detected, strong phenotype).

### The infective cycle of CaLCuV is altered in *CaMV35S-CRL1 *and *CRL5-RNAi *lines

**Figure 5:**
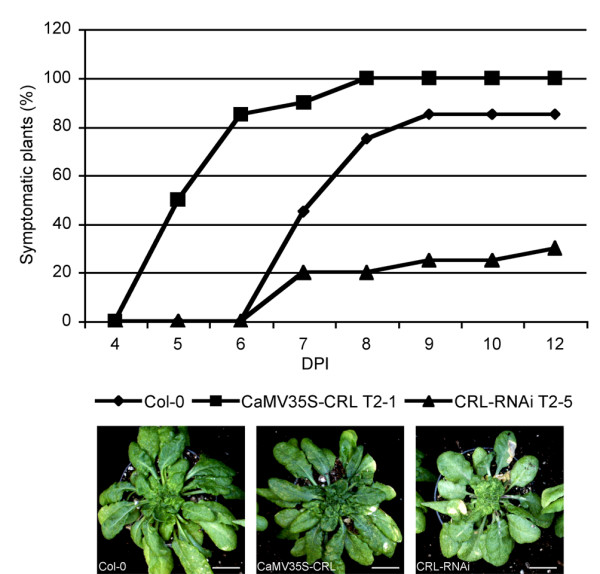
**Infectivity assay of CaLCuV on wild-type (Col O), *CaMV35S-CRL *T2-1 and *CRL-RNAi *T2-5 plants**. After inoculation, plants were evaluated each day for symptom development and the percentage of symptomatic plants was obtained. Thirty plants were analyzed in each case. Typical symptoms (12 dpi) in transgenic and wt plants are shown. Bars = 1 cm

To assess the possible role of *CRL *gene in geminivirus infection cycle, we compared the ability of CaLCuV to infect wt, *CRL *over-expressing and silenced plants. Thirty plants at the 6-8 leaves stage of each type were inoculated on the apical tissue by a biolistic method. After inoculation, plants were evaluated daily for typical symptom expression. As shown in figure 5, the final percentage of plants expressing symptoms upon CaLCuV challenge, evaluated at 12 days after inoculation (dpi), was 30% for *CRL*-*RNAi *plants (T2-5 line), 100% for *CaMV35S-CRL *plants (T2-1 line) and 85% in the case of wt plants. In addition to present the highest inoculation efficiency, CRL over-expressing plants also developed symptoms two days earlier than wt and CRL silenced plants. Since the inoculation efficiency is practically 100% under the conditions used in these experiments, the lack of symptoms is probably due to inefficient replication and/or movement processes in the inoculated plants. Indeed, as mentioned below, viral DNA was detected, although at low concentrations in those symptomless, *CRL*-*RNAi *plants.

Interestingly, although the 3 types of plants showed differences in the efficiency to develop symptoms as well as in the time needed for symptom appearance, the severity of the symptoms induced by CaLCuV was similar at all cases. However it has to be mentioned that *CRL *silenced plants showed a characteristic phenotype.

**Figure 6:**
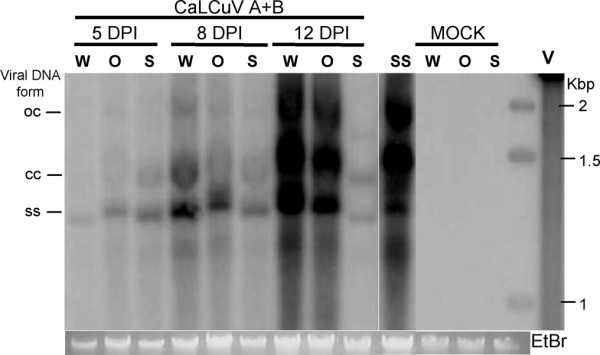
**Southern blot analysis of wild-type and transgenic CalCuV-infected plants**. Total DNA was extracted from a mix of 5 rosettes and loaded in each well of an agarose gel. A fragment of viral component A was used as probe. (W) Wild-type plants, (O) *CaMV35S-CRL *T2-1 plants, (S) *CRL-RNAi *T2-5 plants, (SS) *CRL-RNAi *T2-5 symptomatic plants, (V) viral DNA-A cloned into Bluescript plasmid. Viral forms (open circular viral-DNA, oc; closed circular viral-DNA, cc; single stranded, ss) are indicated. Ethidium bromide staining of genomic DNA is shown as a loading control.

A Southern blot analysis was carried out to compare the concentration of viral DNA as well as the replicative forms found in the 3 types of plants. *CaMV35S-CRL *T2-1, *CRL-RNAi *T2-5 and wild-type plants were inoculated with CaLCuV DNA (both components) by biolistic method and inoculated and systemic leaves (leaves that appeared after the inoculation) were collected at 5, 8 and 12 dpi. Total DNA was extracted and hybridized against a full-length DNA A probe. Southern blot results showed similar geminiviral DNA forms in extracts from all three types of plants indicating that viral replication was carried out in a similar manner (Figure 6). In terms of viral DNA concentration, however, some differences were observed. The hybridization revealed that the viral concentration at 5 dpi, although relatively low, was also very similar in both types of transgenic (over-expressing and silenced) lines and wt plants. As shown in figure 5, at this time point, 50% of the inoculated, *CRL *over-expressing plants had developed symptoms whereas wt and silenced plants have not developed yet any symptoms. At 8 dpi, all *CRL *over-expressing plants were already showing symptoms whereas only 20% of the silenced plants developed symptoms. In the case of the wt, almost 80% of the plants were symptomatic. As shown in figure 6, viral concentration in wt and *CRL *over-expressing plants at 8 dpi were similar whereas silenced plants showed a somewhat reduction of viral DNA concentration.

At 12 dpi, most of the silenced plants remained symptomless and their concentration of viral DNA was greatly reduced compared to the levels found in either, wt or over-expressing plants. Additionally, one of the few silenced plants that developed virus-induced symptoms was also analyzed. The viral DNA levels in this case were similar to the also symptomatic plants from the other two types, wt and over-expressing (Figure 6, lane SS). Although the plants of the silenced line evaluated belong to the T2 generation, it is clear that there is some type of "segregation" or variation of the silencing level, therefore, in those plants in which the silencing is not complete, the virus is able to replicate and move as in a wt plant.

At this moment is not possible to determine if the differences in viral DNA concentration are due to a reduced, virus replication rate, to a less efficient virus movement or a combination of both processes. However, two observations suggest a defect in viral movement. First, the concentrations of viral DNA in the early stage of infection are similar in all 3 types of plants. Second, the concentrations of viral DNA in wt and *CaMV35S-CRL *T2-1 plants were similar at all time points analyzed. This suggests that the differences observed in the CRL over-expressing plants in relation to wt line (early appearance of symptoms and increased percentage of symptomatic plants) were not due to an improved viral replication.

### CaLCuV movement is affected in *CRL-RNAi *T2-5 and supported in *CaMV35S-CRL *T2-1 lines

To confirm that CRL is involved in viral movement, we inserted the ORF for the green fluorescent protein (GFP) into CaLCuV A component generating a CaLCuV:GFP fusion. In this construct, the GFP ORF partially replaced the coat protein (CP) ORF; therefore, its expression will be driven by CP promoter (Figure 1d). It has been reported that CP is not required for CaLCuV replication and movement in Arabidopsis [37]. Wt and both types of transgenic plants were inoculated with a mixture of CaLCuV A-GFP and CaLCuV-B (Figure 1e). Inoculated and systemic leaves (leaves that appeared after the inoculation) were observed with a fluorescence microscope at 5, 8 and 12 dpi using short-wave blue light (460 to 490 nm). Under these conditions, chlorophyll and GFP show a distinguishable fluorescence (red and yellow-green, respectively). Mock and not inoculated plants were used as controls.

GFP fluorescence was clearly detected in all inoculated leaves of the three type of plants analyzed: wt, *CaMV35S-CRL *and *CRL-RNAi*. This confirmed that the modified virus was able to replicate and express GFP. None of the mock-inoculated plants showed GFP fluorescence, confirming that the green fluorescence was indeed due to the presence of the virus fusion.

**Figure 7:**
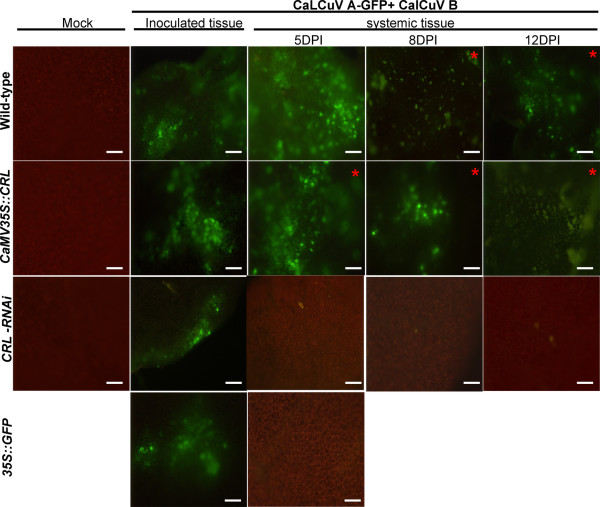
**Fluorescence analysis**. Fluorescence analysis of *CaMV35S-CRL *T2-1, *CRL-RNAi *T2-5 and wild-type plants inoculated with CaLCuV:GFP (both components are present). Representative results from each line and time are shown. Samples taken from symptomatic leaves are marked with asterisk. 35S::GFP was used as a control. GFP expression was observed by using a fluorescent microscope (× 10). Bars = 100 μm.

To verify the long distance movement of the virus in the infected plants, systemic, non-inoculated leaves were also analyzed under the fluorescence microscope. At 5 dpi, inoculated wt plants had not develop symptoms, however, it was possible to detect GFP fluorescence on the systemic leaves analyzed. GFP fluorescence was also detected in the systemic leaves from the inoculated *CaMV35S-CRL *over-expressing plants (Figure 7). It is important to note that in this case, on the contrary to wt plants, the plants had already started to display the symptoms of CaLCuV infection. On the other hand, GFP fluorescence was almost undetectable on the systemic leaves from the *CRL-RNAi *silenced plants; only a few isolated cells displayed a low level of fluorescence. As already mention, at this time period most of the *CRL-RNAi *plants remain symptomless. Similar results were obtained at 8 and 12 dpi. A general observation, however is that intensity of the fluorescence was lower when compared with that observed at 5 dpi (Figure 7). These results confirmed that the CaLCuV movement was affected in the *CRL-RNAi *T2-5 line, suggesting that CRL protein is involved for viral movement mentioned.

## Discussion

Enhancer and gene trap lines have been shown to be useful tools for evaluating gene expression modifications in several stresses and pathogen infections [28]. Trap lines have been also used to identify senescence-associated genes [38], oxygen deprivation-regulated genes [39], genes associated to seed germination [40] and female gametogenesis [41,42]. We report here the use of gene trap lines for the identification of genes whose expression is modified during CaLCuV infection. Although our genetic screen is far from reaching saturation, these results indicate the large potential for the identification of genes that respond to CaLCuV infection following our experimental strategy. To our knowledge, this is the first report that documents the identification of geminivirus-responsive genes using *A. thaliana *gene trap lines.

Using a screen of MGT lines, we have identified genes whose expression is modified upon CaLCuV inoculation. A further characterization of a selected candidate has resulted in the demonstration that the gene *CRUMPLED LEAF *(*CRL*) is involved in the infective cycle of the virus. *CRL *has been previously reported as involved in the morphogenesis of all plant organs and the division of plastids [29]. It was also reported that in a *crl *mutant, the planes of cell division are distorted in shoot apical meristems, root tips and embryos. In addition, the mutant is dwarf and present pale green and crumpled leaves. CRL protein was observed associated with plastid membranes and, more recently, it has been shown that a *crl A. thaliana *mutant present cells without detectable plastids [29,31]. Although CRL protein is conserved in various species of dicots, monocots and cyanobacterias, no similarity to proteins with predicted or known function has been reported.

The usefulness of gene trap technology to identify genes responsive to viral infections is additionally supported by the fact that the *CRL *gene was not identified in screenings designed to detect genes regulated during different virus infections (including CaLCuV) using *A. thaliana *microarrays and sDNA-AFLP analysis (AffyID 24849_at; gene At5 g51020) [25,43-45]. The variety of results observed in the screening also suggests that viral induction of some genes can be a highly localized process (in time or space), thus, those genes could be easily missed in analysis with some methodologies (microarrays, differential libraries) due to a dilution of the mRNAs or an inappropriate timing for sample collection.

Viruses can alter the transcriptional networks of their host [18,46]. In the case of Arabidopsis, changes in host gene expression have been documented in different stages of the infection by several types of viruses including caulimoviruses (CaMV), cucumoviruses (CMV), tobamoviruses (TVCV), potexviruses (PVX), potyviruses (PVY), and more recently by geminiviruses [44,47,48] with a wide array of cellular processes that likely reflect the biochemical and physiological changes involved in the development of the disease syndrome. As mentioned before, none of those reports included *CRL *as a virus-induced gene.

The expression of *CRL *in CaLCuV-infected plants shows an interesting pattern. In terms of the type of response, a short but strong induction, it resembles the one observed with early response genes associated to WRKY-type transcription factors [49]. However, early response is usually observed in a matter of minutes, not days as observed with *CRL*. Consistently, an analysis of the *CRL *promoter did not revealed any WRKY (TTGAC) boxes. In terms of timing (3-5 days after inoculation), the response associated with the one reported for PR-1, a common molecular marker for systemic acquired resistance (SAR) [16,17]. However, PR-1 expression, unlike *CRL *remains high for a longer period with a plateau-type of response whereas *CRL *expression sharply goes down to basal levels around 7 dpi.

Silencing and over-expressing a gene are common strategies to study its function. In the case of *CRL*, its constitutive expression did not produce an informative phenotype (Figure 3b and 3g) even though the levels of *CRL *transcript were higher than the levels observed in wt plants. In general, the plants were basically similar to wt although some plants showed larger size than the wt. The lack of a phenotype after over-expressing a single gene is not uncommon and it has been suggested that the plasticity of the plant metabolism can balance most cases of ectopic expression. Nevertheless, the over-expressing lines did show some differences in the inoculation experiments since they developed symptoms 2-3 days earlier than wt plants. This enhanced susceptibility was also reflected in the percentage of inoculated plants that became infected. This suggested that CRL might somehow facilitate the establishment of the infection or the spread of the CaLCuV. At this point, it is not possible to differentiate which of the basic processes in an infective cycle, replication or movement, is affected in the over-expressing lines.

In contrast to the results obtained with the over-expressing lines, a gradient of phenotypes were observed in the silenced, *CRL-RNAi *lines. The strongest phenotype was similar to the one observed by Asano et al. [29,31]. This phenotype resembles the symptoms observed in a viral infection (crumpling and deformation of leaves). However, it has to be mentioned that in the case of CaLCuV-infected Arabidopsis, there were noticeable differences between the phenotype of the silenced plant and the one observed in a CaLCuV-infected one. In this second case, the severity of the symptoms was stronger and plant development was affected resulting in a small size. The range of phenotypes observed in the silenced lines has been observed in many systems targeting endogenous genes and it has been attributed to the differences in the silencing efficiency [32-35,41,42].

In addition to the phenotype observed in the silenced lines, these plants also presented some differences with wt plants when challenged with CaLCuV. The time for symptom appearance was basically similar in both cases, however, the number of CRL-silenced plants that became infected was considerably lower than the number observed for wt and over-expressing lines. By 12 dpi, only 30% of the inoculated plants showed symptoms whereas in the case of wt or over-expressing lines the percentage obtained was dramatically higher (80-100%). In many cases, a brief delay in symptom appearance or lower infection efficiency has been reported as a degree of resistance, or tolerance to viral infection. Therefore, the lack of CRL protein confers an interesting characteristic for biotechnological developments, although it applicability is currently limited due to the phenotype showed by the silenced lines.

Southern blot analysis demonstrated that neither the lack of *CRL *completely prevents CaLCuV replication nor does its over-expression result in an increased viral replication. In the first case, viral replicative forms were still detected, although at a lower concentration, in the symptomless plants (70% of the inoculated plants) at 12-15 dpi. Systemic tissue analyzed by PCR at 30 dpi shown no viral DNA. This suggested that CaLCuV is still able to replicate and move, although the overall infective process seems highly hindered. It has to be mentioned that the plants that did develop symptoms (30%) did show viral DNA concentration equivalent to that observed in wt plants. In the case of the over-expressing plants, viral DNA concentration was also similar to the one found in infected wt plants suggesting that in this case the ectopic expression of CRL protein does not affect virus cycle.

These results and the fact that CRL has been reported as a membrane protein suggested its possible involvement in facilitating the movement of the virus. To evaluate this hypothesis, viral movement analyzes were carried out using a CaLCuV A-GFP construct. In the case of the *CRL*-silenced plants fluorescence spots were observed solely at inoculation sites indicating a deficient movement of the modified virus in those plants. On the other hand, GFP fluorescence was observed in systemic tissues in both *CaMV35S-CRL *and wt plants, although it was common to detect GFP fluorescence a day earlier in the case of the over-expressing plants.

## Conclusion

In order to carry out a successful infection, a virus must spread between cells moving from their replication sites at cell periphery and then traverse intercellular channels to enter the neighbouring cell until the vascular system is reached for its long-distance transport. Cell-to-cell transport of most plant viruses is mediated by specific virally encoded factors termed movement proteins (MPs). However, most of the cell-to-cell transport machinery is provided by the host cell [7,50]. Many host plant proteins that bind viral MPs have been identified [51] and several of them have been shown to influence viral movement [52-57]. Geminiviruses, and other DNA viruses, might have some particular differences in the mechanisms to spread throughout an infected plant when compared to RNA viruses. A major difference is the nuclear replication, although the nature of the genome itself (RNA vs. DNA) might have influence also [4]. Several studies using the two-hybrid system have provided evidence of interactions between geminivirus movement proteins (MP and NSP) and host proteins [58,59]. It is not clear at the moment, if CRL protein interacts with either one or both of the geminiviral proteins MP and NSP. Alignment analysis of *CRL *sequence with its homologues in others plants indicates the presence of one putative trans-membrane domain localized between amino acids 16-21. CRL protein fused to GFP was localized mainly in chloroplast [29]. However, its association with other membranes cannot be discarded.

Based on the characteristics of CRL protein and the data with CRL-silenced and CRL-expressing transgenic plants, several possibilities for the involvement of CRL in the geminivirus infective cycle can be envisioned. First, although CRL has been reported as an outer envelope membrane protein, it does not present an obvious transmembrane-chloroplast domain [29,31]. It is possible that CRL could also be localized on the plasmatic membrane, specifically in the plasmodesmata vicinity where, throughout an interaction with viral proteins (e.g., MP) could participate in viral transport [4].

Second, geminivirus might have evolved to adapt themselves to transport and/or communication pathways important for plant metabolism, therefore, it is feasible that viral movement can be affected by changes in cellular metabolism, such as the ones occasioned by the modification of *CRL *expression. For example, it has been suggested that the envelope membrane of plastids is the site of transport and exchange of ions and metabolites [60,61]. The lack of CRL protein, then, could affect the efficiency of processes such as the import of nuclear proteins involved in generation of metabolites necessary for plant morphogenesis or even plastid division [29,31]. Those altered processes, in turn, could affect virus cycle directly or indirectly by affecting other important cellular process in which the virus relies to for its own transport. Therefore, the effect on virus replication/movememnt observed in CRL-silenced plants (important reduction but not total block of replication) is likely to be a secondary effect after plastid metabolism disruption.

It is clear, in any case, that further investigations are required to elucidate: a) the precise molecular function of CRL protein on geminiviral infection, b) the disease-like phenotype observed in CRL-silenced plants, and c) if a possible interaction between CRL and geminiviruses exists because of the ancestral prokaryotic characteristic of both, plastids [62,63] and geminiviruses [64-67].

## Methods

### Plant material and growth conditions

A collection of Enhancer- (MET) and Gene-Trap (MGT) lines were generated in the Laboratory of Reproductive Development and Apomixis, Cinvestav-Irapuato [42]. The lines were selected in Murashige and Skoog (MS) medium containing 50 μg/ml kanamycin. Primary transformant seedlings CaMV35S-CRL and CRL-RNAi lines were selected using 0.05% of BASTA herbicide. Subsequent transformant generations were selected in MS medium containing 50 or 10 μg/ml glufosinate ammonium (Crescent chemical, Islandia, NY). After germination, seedlings were grown on 3:1:1 Mix3-Sunshine (SunGro, Bellevue, WA), vermiculite, and perlite (vol/vol/vol ratio) containing 1.84 Kg/m3 of 14-14-14 slow-release fertilizer (Osmocote, Sierra, Marysville, OH) in a controlled environment chamber at 22°C with a photoperiod of 8 h of day and 16 h of dark.

### DNA Isolation and TAIL-PCR

Total DNA was isolated by grinding inoculated tissue (6-8 leaves by plant) in liquid nitrogen in presence of buffer CTAB [68]. For TAIL-PCR 5 ng of total DNA was used to amplify the tagged sequences using the program and primers described elsewhere [30].

### Generation of CaLCuV A-GFP, RNAi, over-expressing, pCRL::UidA constructs and transgenic plants

To generate the CaLCuV A-GFP, GFP gene was digested from pCAT GFP [69] and cloned into pCPCbLCVA.007 [37] between *Xho*I and *Bgl*II restriction sites (Figure 1d). In this construct the GFP gene is under the direction of the CP promoter.

To generate the *CaMV35S-CRL *and *CRL-RNAi *lines, we amplified a cDNA corresponding to CRUMPLE LEAF gene (Accession no. At5 g51020) by RT-PCR, using the following primers, CRL-sense 5'-CG**TCTAGAGGCGCGCC**ATGGGTACCGAGTCGGGT-3' (restriction sites XbaI and AscI in boldface) and CRL-antisense 5'-CG**GGATCCATTTAAAT**CTAGTCTTGCAAGATGAG-3' (restriction sites BamHI and SwaI are shown in boldface). *CRL *cDNA was cloned into TOPO-PCRII (Invitrogen) and correct insert orientation was selected by restriction analysis (resulting in pCRL-TOPO). For *CRL-RNAi *construct, we digested pCRL-TOPO with *BamH*I to excise the *CRL *fragment to be cloned into pFGC5941 vector (Figure 1a); the sense orientation construct (pre *CRL-RNAi*) was selected by restriction analysis [35]. To add the antisense *CRL*, pCRL-TOPO was digested with *Asc*I and *Xho*I and the *CRL *fragment was subcloned into corresponding sites of pre *CRL-RNAi *plasmid (Figure 1b). To generate the *CaMV35S-CRL *(Figure 1c), the *CHSA *intron of pFGC5941 (Figure 1a) was replaced by the *CRL *fragment obtained from pCRL-TOPO digested with *Asc*I and *Xba*I. To generate the pCRL::UidA, we amplified 983 bp of *CRL *promoter sequence with the followings primers, pCRL-sense 5'-GGG**AAGCTT**TCAGCAGAAGATG-3' (restriction site HindIII in boldface) and pCRL-antisense TC**TCTAGA**GTGAGAGAACGAG (restriction site XbaI in boldface). The promoter fragment was cloned into TOPO-PCRII (Invitrogen) and subcloned into pBI 101.2 vector. The *CRL-RNAi, CaMV35S-CRL *and pCRL::UidA constructs were introduced into *A. tumefaciens *PGV2260, which was then used to transform *A. thaliana *(Col-0) by floral dip [70].

### Analysis of GUS activity

GUS activity was assayed by histochemical staining as described in [71]. Chlorophyll was cleared incubating the leaves in Methanol-acetone (1:1 vol/vol) and imaged on a Leica stereoscope.

### Viral Inoculation

Plants at the six- to eight-leaves stage were inoculated by biolistic delivery with a modified handheld, low-pressure apparatus that allowed the targeting of a specific tissue (leaf, rosette or apex). Individual leaves of transposant plants were directly shot at 50 psi helium pressure with golden particles (0.5 Micron, BioRad) covered with 200 ng of each viral DNA (pCPCbLCVA.007 and pCPCbLCVB.002, [37]); kindly provided by D. Robertson, North Carolina State University) or with pBluescript for mock-inoculations. For virus movement and symptom development experiments, 20 plants (Figure 1e) of each line were inoculated at the same conditions.

### RNA Isolation and Real-time PCR

Total RNA was isolated grinding five rosettes (8-12 leaves) from each time point (1, 3, 5, and 7 dpi) in liquid nitrogen in the presence of TRIzol reagent (Invitrogen Life Technologies). One μg of total RNA was treated with amplification-grade DNAse I (Life technologies) for 20 min; after heat inactivation, 500 ng was used to generate cDNA with Superscript II reverse transcriptase (Life technologies) using oligo (dT)_18_.

Real-time PCR for relative quantification was performed in 20-μl reactions using 0.5 μl of cDNA (12.5 ng) along with 1 μl of each 10 μM primer and 10 μl of 2× Platinum SYBR Green qPCR Super Mix UDG (Life technologies) in a BioRad iCycler thermocycler. The following primers were used for specific *CRL *cDNA amplification: QRTCRL-sense (5'-GATCCTGATAATTACTTCAAC'), QRTCRL-antisense (5'-AACGGTTTCTGAGGAGTCCT-3'), AL2-sense (5'-ACTACCAAAAATGCAAAATT-3'), AL1-antisense (5'-CAGCTCTCCTTTTAGCAATC-3'). To normalize data, parallel amplifications of 16S rRNA were done as internal reference with primers Actin-sense (5'-TCCCTCAGCACATTCCAGCAG-3') and Actin-antisense (5'-AACGATTCCTGGACCTGCCTC-3'). Data analysis was performed as described before [72].

### Southern blot analysis

Inoculated (6-8 leaves by plant) and systemic (4-6 leaves by plant) tissue from six infected plants was sampling. Total DNA was extracted as described by earlier [73]. Ten micrograms of total DNA per well was loaded in a 1% agarose gel. After electrophoresis, DNA was transferred to Hybond N^+ ^membrane (Amersham Biosciences, Piscataway, NJ) by capillary action with a 10× solution of 0.15 M NaCl plus 0.015 M sodium citrate (SSC), and UV-crosslinked. The fragment (1090 bp) obtained after digestion with XhoI and NcoI of pCPCbLCVA.007 [37] was used as probe. Probe was radioactively labelled using Redi prime II Kit and (α-^32^P) dCTP (Amersham). Hybridization was performed overnight at 65°C as described in Rapid-hyb Kit protocol (Amersham). Membrane was then washed twice in 2× SSC, 1% sodium dodecyl sulphate (SDS), and twice in 0.2× SSC, 1% SDS, all at 65°C. Hybridization signals were detected on a phosphorimager system.

### Northern blot and small RNAs detection

For northern blot analysis, total RNA was extracted from rosette (8-12 leaves stage) using Trizol (Invitrogen) and following the manufacturer's instructions. Ten micrograms of RNA were separated in a 1.3% agarose gel containing 17% formaldehyde and blotted onto hybond N^+ ^membranes. Membrane was cross-linked using UV irradiation and hybridized at 65°C as described.

For small RNA analysis, total RNA from rosette leaves were enriched for low molecular weight RNAs using Hamilton's homogenization solution [74]. Low molecular weight RNA was normalized by spectrophotometry to 100 μg/lane, separated by electrophoresis through 15% polyacrylamide, 7 M urea, and transferred to Zeta-Probe GT membranes (Bio-Rad). Membrane was cross-linked using UV irradiation and hybridized at 50°C as described by [75].

### GFP fluorescence analysis

GFP expression was monitored by using a fluorescence stereoscope (Leica MZ8) with the set of filters GFP-Plus (excitation filter: 480/40 nm; emission: 510 nm) and a fluorescence microscope (Leica DMRE) with an excitation filter (I3) BP 450-490 nm, a dichroic mirror at 510 nm and an observation filter at 515 nm. Images were generated by using a digital camera (Spot Diagnostic Instruments) and edited by using Adobe Photoshop CS software.

## Competing interests

The authors declare that they have no competing interests.

## Authors' contributions

DLTS carried out the experimental work, participated in the analysis and design of the project and prepared initial versions of figures and manuscript. JPVC was responsible of the design, generation and characterization of the transposant lines. RFRB was responsible for all virological aspects of the experimental study, participated in its design, and coordinated the project. All authors read and approved the final manuscript.
